# Hyperactivity/restlessness is associated with increased functional connectivity in adults with ADHD: a dimensional analysis of resting state fMRI

**DOI:** 10.1186/s12888-019-2031-9

**Published:** 2019-01-25

**Authors:** Peter Sörös, Eliza Hoxhaj, Patricia Borel, Chiharu Sadohara, Bernd Feige, Swantje Matthies, Helge H. O. Müller, Katharina Bachmann, Marcel Schulze, Alexandra Philipsen

**Affiliations:** 1 Psychiatry and Psychotherapy, School of Medicine and Health Sciences, University of Oldenburg, Oldenburg, Germany; 2 Research Center Neurosensory Science, University of Oldenburg, Oldenburg, Germany; 3 Department of Psychiatry and Psychotherapy, Medical Center - University of Freiburg, Faculty of Medicine, University of Freiburg, Freiburg, Germany; 4 Department of Psychiatry and Psychotherapy, University of Bonn, Bonn, Germany

**Keywords:** ADHD, Adult, Resting state fMRI, Functional connectivity, Inattention, Hyperactivity, Impulsivity, Age

## Abstract

**Background:**

Adult attention-deficit/hyperactivity disorder (ADHD) is a serious and frequent psychiatric disorder of multifactorial pathogenesis. Several lines of evidence support the idea that ADHD is, in its core, a disorder of dysfunctional brain connectivity within and between several neurofunctional networks. The primary aim of this study was to investigate associations between the functional connectivity within resting state brain networks and the individual severity of core ADHD symptoms (inattention, hyperactivity, and impulsivity).

**Methods:**

Resting state functional magnetic resonance imaging (rs-fMRI) data of 38 methylphenidate-naïve adults with childhood-onset ADHD (20 women, mean age 40.5 years) were analyzed using independent component analysis (FSL’s MELODIC) and FSL’s dual regression technique. For motion correction, standard volume-realignment followed by independent component analysis-based automatic removal of motion artifacts (FSL’s ICA-AROMA) were employed. To identify well-established brain networks, the independent components found in the ADHD group were correlated with brain networks previously found in healthy participants (Smith et al. PNAS 2009;106:13040–5). To investigate associations between functional connectivity and individual symptom severity, sex, and age, linear regressions were performed.

**Results:**

Decomposition of resting state brain activity of adults with ADHD resulted in similar resting state networks as previously described for healthy adults. No significant differences in functional connectivity were seen between women and men. Advanced age was associated with decreased functional connectivity in parts of the bilateral cingulate and paracingulate cortex within the executive control network. More severe hyperactivity was associated with increased functional connectivity in the left putamen, right caudate nucleus, right central operculum and a portion of the right postcentral gyrus within the auditory/sensorimotor network.

**Conclusions:**

The present study supports and extends our knowledge on the involvement of the striatum in the pathophysiology of ADHD, in particular, in the pathogenesis of hyperactivity. Our results emphasize the usefulness of dimensional analyses in the study of ADHD, a highly heterogeneous disorder.

**Trial registration:**

ISRCTN12722296 (10.1186/ISRCTN12722296).

**Electronic supplementary material:**

The online version of this article (10.1186/s12888-019-2031-9) contains supplementary material, which is available to authorized users.

## Background

Attention-deficit/hyperactivity disorder (ADHD) is a common and impairing psychiatric disorder characterized by varying degrees of inattention, hyperactivity, and impulsivity. ADHD is not limited to children and adolescents. In 40–60% of children with ADHD, the disorder persists into adulthood [1]. In adults with ADHD, inattention may present as a lack of concentration, forgetting appointments, and a failure to plan and organize tasks, while hyperactivity may be experienced as restlessness and difficulty in relaxing [1, 2]. ADHD symptoms frequently lead to problems at school and at the workplace as well as to difficulties with social interaction and relationships [2].

The pathogenesis of ADHD is incompletely understood. Clinical, genetic and experimental evidence suggests that ADHD is a multifactorial disorder, associated with neurochemical [3], anatomical [4–6] and functional [7, 8] changes of neuronal networks. The notion that ADHD is, in its core, a disorder of dysfunctional brain connectivity within and between several neurofunctional networks has gained wide acceptance [8, 9].

One way to investigate the circuitry of the brain is to perform resting state functional magnetic resonance imaging (rs-fMRI). In contrast to task-based fMRI, rs-fMRI measurements record the spontaneous fluctuations of brain activity during wakeful rest, i.e. in the absence of an experimental task or stimulation. Using rs-fMRI, several distinct resting state networks (RSNs) have been identified in health and disease [10].

Both in children and adults with ADHD, a dysfunction of the default mode network (DMN) has been postulated. The DMN is a set of brain regions, including the posterior cingulate cortex, precuneus, and medial prefrontal cortex, which are active during rest and become deactivated with the initiation of a task [11, 12]. A pioneering rs-fMRI study on 20 ADHD adults (mean age: 34.9 years; 16 men) and 20 healthy participants (mean age: 31.2 years; 14 men) found decreased functional connectivity within the DMN and between posterior regions of the DMN (i.e., the precuneus and posterior cingulate) and the dorsal anterior cingulate [13].

Since then, a large number of studies on rs-fMRI in individuals with ADHD have been published [8]. Most of these studies have investigated children and adolescents and performed categorical analyses, comparing functional connectivity between individuals with ADHD and healthy controls. Several research groups have used the freely available ADHD-200 sample, consisting of rs-fMRI data of 285 children and adolescents with ADHD and 491 healthy age-matched controls [14]^1^ to address differences in functional connectivity in categorical and dimensional analyses [15–17].

Our primary interest lies in adults with ADHD [18, 19]. For this population, only a relatively small number of studies on resting state functional connectivity is available. These studies confirmed and extended our pathophysiological knowledge of adult ADHD. However, most studies investigated adults who have received methylphenidate (for notable exceptions, see [20]). In addition, most studies on adult ADHD performed categorical comparisons between individuals with ADHD and controls. As ADHD is a disorder of remarkable clinical heterogeneity, we decided to investigate functional connectivity within resting state networks in relation to ADHD symptom severity in a group of methylphenidate-naïve adults with childhood-onset ADHD following a dimensional approach to investigate psychiatric disorders [21].

To study functional connectivity within neural networks, we performed a group independent component analysis (ICA) with dual regression. The group ICA identifies a set of independent component maps that are common to our entire sample. Dual regression is a mathematical approach that uses these independent component maps as network templates to identify the corresponding functional connectivity maps, indicating the strength of functional connectivity in each subject (for a detailed explanation, see [22]).

## Aim and hypotheses

The aims of the present study are twofold. First, we will characterize well-established resting state networks, previously described in healthy individuals, in our sample of adults with ADHD. Second, we will investigate potential associations between the functional connectivity in these networks and the individual severity of core ADHD symptoms (inattention, hyperactivity, and impulsivity). We hypothesized that functional connectivity within the default mode network will be smaller in individuals with ADHD with increased symptom severity.

## Methods

### Participants

This study is part of a larger project on structural and functional changes of the brain in adults with ADHD. Structural MRIs from this project were analyzed using surface-based morphometry [23]. Results of task-related fMRI measurements were reported by Bachmann et al. [24].

Resting state fMRI data sets from 59 adults with ADHD were analyzed for this study. Data sets were taken from a randomized controlled trial that compared the efficacy of a mindfulness training program (mindfulness awareness practice) to an active control condition (structured psychoeducation) in adult ADHD [25]. A control group of healthy individuals was therefore not studied. All datasets analyzed here were recorded at baseline, i.e. before the mindfulness training program or psychoeducation started.

**Fig. 1:**
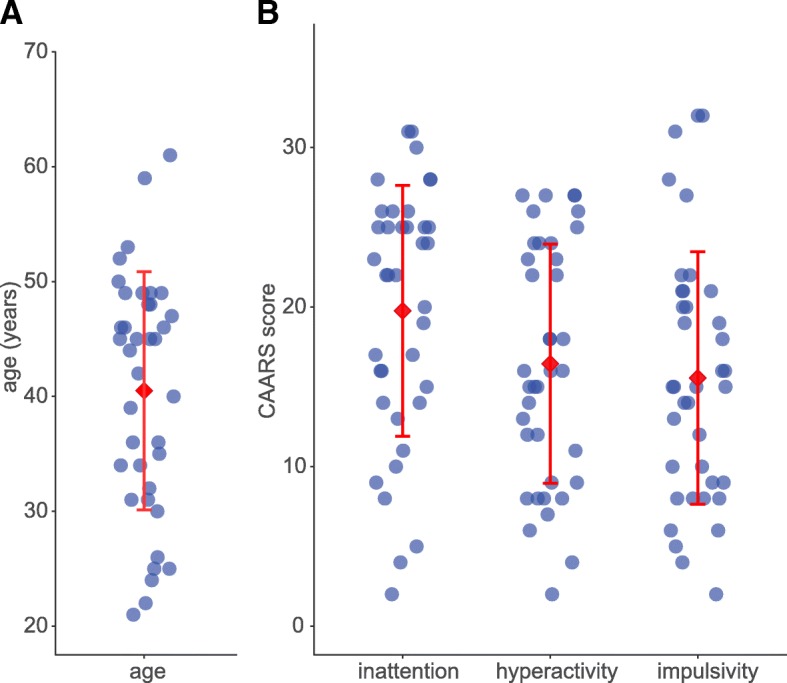
1**a**. The distribution of age in the analyzed sample of 38 adults with ADHD. **1b**. The distribution of the scores on the CAARS inattention/memory problems, hyperactivity/restlessness, and impulsivity/emotional lability subscales. The red diamond represents the mean, the error bars the standard deviation in both parts of the figure

After head motion correction using FSL’s MCFLIRT [26], 21 individuals were excluded because the maximum absolute displacement was > 1.5 mm (half of the isotropic voxel size). The absolute displacement, as determined by MCFLIRT, summarizes translation and rotation across all three axes for every volume relative to the middle volume of the rs-fMRI data set [27]. This criterion was chosen according to the rs-fMRI study by Mostert et al., investigating adults with ADHD using FSL’s dual regression approach [28]. Thus, the data sets of 38 individuals with ADHD (20 women) were included in the final analysis. Demographics and clinical characteristics of this sample are summarized in Table 1. The distribution of participants’ age is illustrated in Fig. 1a.

**Table 1:** Demographics and clinical characteristics of all adults with ADHD included in the final data analysis

Age	
Mean ± standard deviation (range)	40.5 ± 10.4 years (21–61 years)
Gender	
Women	20 (52.6%)
Men	18 (47.4%)
Education	
Secondary school (until grade 9)^1^	6 (15.8%)
Secondary school (until grade 10)^2^	11 (28.9%)
High school diploma (until grade 12 or 13)^3^	15 (39.5%)
University degree	6 (15.8%)
CAARS (observer-rated)	
Mean ± standard deviation (range)	
Inattention/memory problems	19.8 ± 7.9 (2–31)
Hyperactivity/restlessness	16.4 ± 7.5 (2–27)
Impulsivity/emotional lability	15.6 ± 7.9 (2–32)
ADHD subtype	
Combined	31 (81.6%)
Inattentive	7 (18.4%)
Co-morbidities: current axis I disorders	
Minor depressive disorder	20 (52.6%)
Anxiety disorder	7 (18.4%)
Obsessive-compulsive disorder	2 (5.3%)
Co-morbidities: lifetime axis I disorders	
Substance dependence	7 (18.4%)
Eating disorder	4 (10.5%)
Co-morbidities: lifetime axis II disorders	
Avoidant personality disorder	5 (13.2%)
Obsessive-compulsive personality disorder	2 (5.3%)
Dependent personality disorder	1 (2.6%)

German: ^1^*Hauptschulabschluss*, ^2^*Realschulabschluss*, ^3^*Abitur*

The clinical trial is registered in the ISRCTN registry^2^ (ISRCTN12722296)^3^ and has been approved by the Ethics Committee of the Faculty of Medicine, University of Freiburg, Germany. All participants provided written informed consent.

Recruitment of participants has been described previously [23, 24]. In brief, participants were recruited at the Department of Psychiatry and Psychotherapy, Medical Center - University of Freiburg, Germany and through ADHD support groups. Inclusion and exclusion criteria have also been described previously [23, 24]. In brief, we included individuals between 18 and 65 years with childhood-onset ADHD, who never used methylphenidate.

### Diagnosis and clinical data

The diagnosis of ADHD was performed by experienced psychiatrists following DSM-IV criteria as described previously [23–25]. ADHD symptom severity was measured with the Conners Adult ADHD Rating Scales [29] in the German long version with 66 items [30]. We present the blind observer-rated CAARS scores (CAARS-O:L) on the inattention/memory problems, hyperactivity/restlessness, and impulsivity/emotional lability subscales. The distribution of the individual scores on the 3 subscales is illustrated in Fig. 1b. Psychiatric comorbidities were assessed using the German version of the Structured Clinical Interview for DSM-IV (SKID) [31].

### MRI data acquisition

Structural and functional images of the brain were acquired on a 3 Tesla Siemens Magnetom Trio with a 12-channel head coil at the Freiburg Brain Imaging Center as described earlier [23, 24]. In brief, a T1-weighted image was acquired using a three-dimensional MP-RAGE sequence with a voxel size of 1 × 1 × 1 mm^3^. For the resting state measurement, T2*-weighted BOLD images were obtained with a voxel size of 3 × 3 × 3 mm^3^ and 36 axial slices with a slice thickness of 3 mm (TR = 2250 ms, TE = 30 ms, no in-plane acceleration, 230 brain volumes, time of acquisition: 8:42 min). The field of view covered the entire cerebrum, but only the most rostral parts of the cerebellum in most participants. All participants were instructed to lie quietly and keep the eyes closed without falling asleep. The resting state measurement was preceded by the T1-weighted image and 2 runs of a 1-back working memory task (time of acquisition: 6:16 min each) and 2 runs of a stop signal task (time of acquisition: 6:09 min each). These task-based fMRI measurements were not included in the present study. The results of the 1-back working memory task have been reported by Bachmann et al. [24].

### Preprocessing of fMRI data

Preprocessing of resting state FMRI data was carried out using FMRIB’s Software Library (FSL, version 5.09)^4^ [32–34]. Preprocessing included removal of the first 5 volumes to allow for signal equilibration (225 volumes were retained) and head motion correction by volume-realignment to the middle volume using MCFLIRT [26]. Brain extraction was performed using BET [35]. Spatial smoothing with a Gaussian kernel of 6 mm full width at half maximum (FWHM) and grand-mean intensity normalization of the entire dataset by a single multiplicative factor were also done.

After performing standard data preprocessing, without temporal filtering, independent component analysis-based automatic removal of motion artifacts (FSL’s ICA-AROMA version 0.3 beta)^5^ was used to identify and remove motion-related ICA components from fMRI data. Here, the ‚non-aggressive‘ option was used, performing a partial component regression. ICA-AROMA carries out probabilistic ICA of individual subjects’ rs-fMRI data using multivariate exploratory linear decomposition into independent components (FSL’s MELODIC, version 3.14) [36], employs four theoretically motivated temporal and spatial features to select motion-related components from MELODIC’s output and finally removes these components from the initial data set through an ordinary least squares regression using FSL’s *fsl_regfilt* command [37]. ICA-AROMA is an effective strategy for removing motion-related artifacts from rs-fMRI data, preserving signal of interest and increasing the reproducibility of resting state networks [38, 39]. ICA-AROMA does not require study-specific training (i.e. manual classification of artifact- and non-artifact-related independent components) and is thus a robust and generalizable approach.

The de-noised data sets were then high-pass filtered with a cutoff of 150 s (0.007 Hz). Registration of functional to high resolution structural images was carried out using boundary-based registration [40] in FLIRT [26]. Registration from high resolution structural to Montreal Neurological Institute (MNI152) standard space was further refined using 12-parameter affine transformation and non-linear registration with a warp resolution of 10 mm in FNIRT.^6^

### Identification of resting state networks

To identify RSNs common to adults with ADHD, all data sets (*n* = 38, preprocessed and de-noised with ICA-AROMA as described above) were concatenated in temporal order to create a single data set. This concatenated data set was then decomposed into 20 spatially independent components using group ICA with MELODIC. A low-dimensional decomposition was chosen to facilitate the comparison of RSNs in adult ADHD with those identified in healthy adults [10, 41, 42]. These 20 components will be used as template maps for dual regression (see next section).

To investigate the occurrence of previously described RSNs in adult ADHD, a spatial cross-correlation between these 20 independent components in our sample and the 20 independent components identified previously [10] was calculated using FSL’s *fslcc* command. MR image files of Smith et al.’s template networks [10] are available for download.^7^ For further analysis and visualization (Fig. 3), 10 canonical RSNs in our data were chosen that showed a high spatial correspondence (> 0.4) with the well-established RSNs published by Smith et al. [10].

### Statistical analysis of resting state networks

To investigate the associations between RSNs and clinical data (sex, age, and ADHD symptom severity), FSL’s *dual_regression* script (version 0.5) was used [22, 43]. In the first stage of dual regression, the full set of 20 template maps (the 20 independent components identified by group ICA) was regressed against each participant’s 4-dimensional rs-fMRI data set, resulting in 20 time series per participant, one for each template map. In the second stage of dual regression, the component-specific time series were variance-normalized and regressed against each participant’s rs-fMRI data set to identify participant-specific spatial maps corresponding to the 20 template maps.

To identify differences between women and men within the 10 canonical networks, a voxel-wise two-sample unpaired t-test with age as regressor of no interest was performed on the participant-specific spatial maps for each network using a general linear model. To identify associations between age and functional connectivity within the 10 canonical networks, age was used as regressor of interest with sex as regressor of no interest in the general linear model. To identify associations between ADHD symptom severity and functional connectivity within the 10 canonical networks, the individual scores on the inattention/memory problems, hyperactivity/restlessness, and impulsivity/emotional lability CAARS subscales were used as separate regressors of interest with sex and age as regressors of no interest. For non-parametric permutation testing, FSL’s *randomise* (version 2.9) was used with 5000 permutations [44, 45]. Statistical thresholding was performed with FSL’s threshold-free cluster enhancement (TFCE) [46] and a family-wise error rate (FWE) of p smaller than 0.05. As the existing literature does not support specific hypotheses regarding the association between whole-brain networks and symptom severity in adult ADHD, this study needs to be exploratory. We decided not to perform correction for multiple comparisons (e.g. Bonferroni correction). To reduce the risk of false positive activation, we only accepted clusters larger than 100 voxels.

## Results

### Head motion

**Fig. 2:**
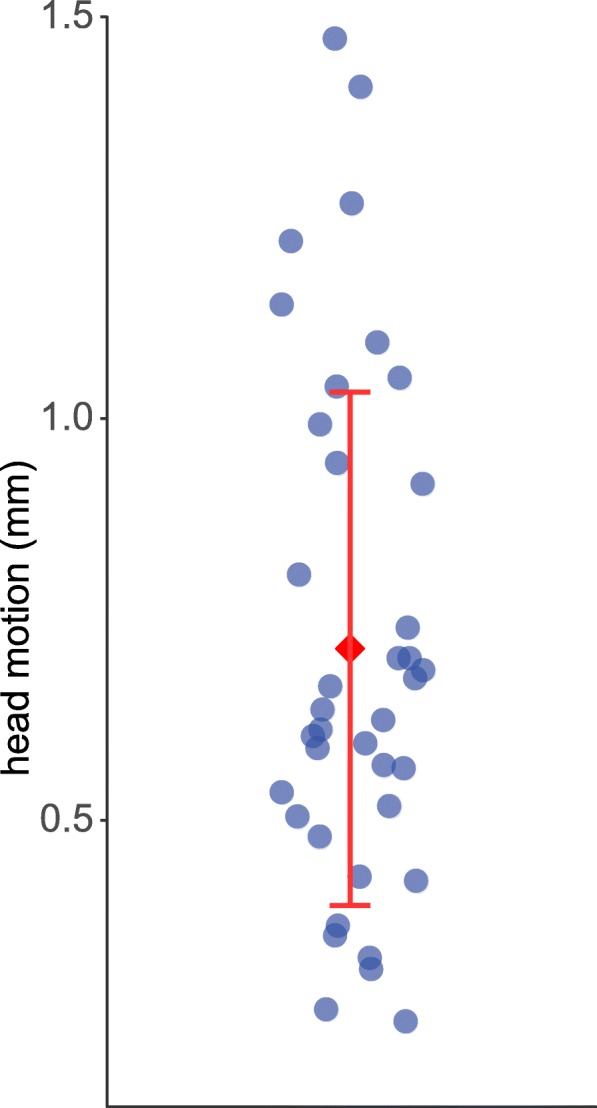
Maximum head motion (absolute displacement) for all 38 adults with ADHD. The figure shows the maximum value of absolute displacement (mm), which summarizes translation and rotation across all three axes for every volume relative to the middle volume of the rs-fMRI data set

Figure 2 shows maximum head motion for every participant, expressed as absolute displacement (relative to the middle volume of the data set) and estimated by FSL’s MCFLIRT. Across all participants, mean maximum head motion was 0.71 mm (SD: 0.32 mm). In the majority of participants, maximum head motion was smaller than 1 mm.

### Resting state networks in adult ADHD

**Fig. 3:**
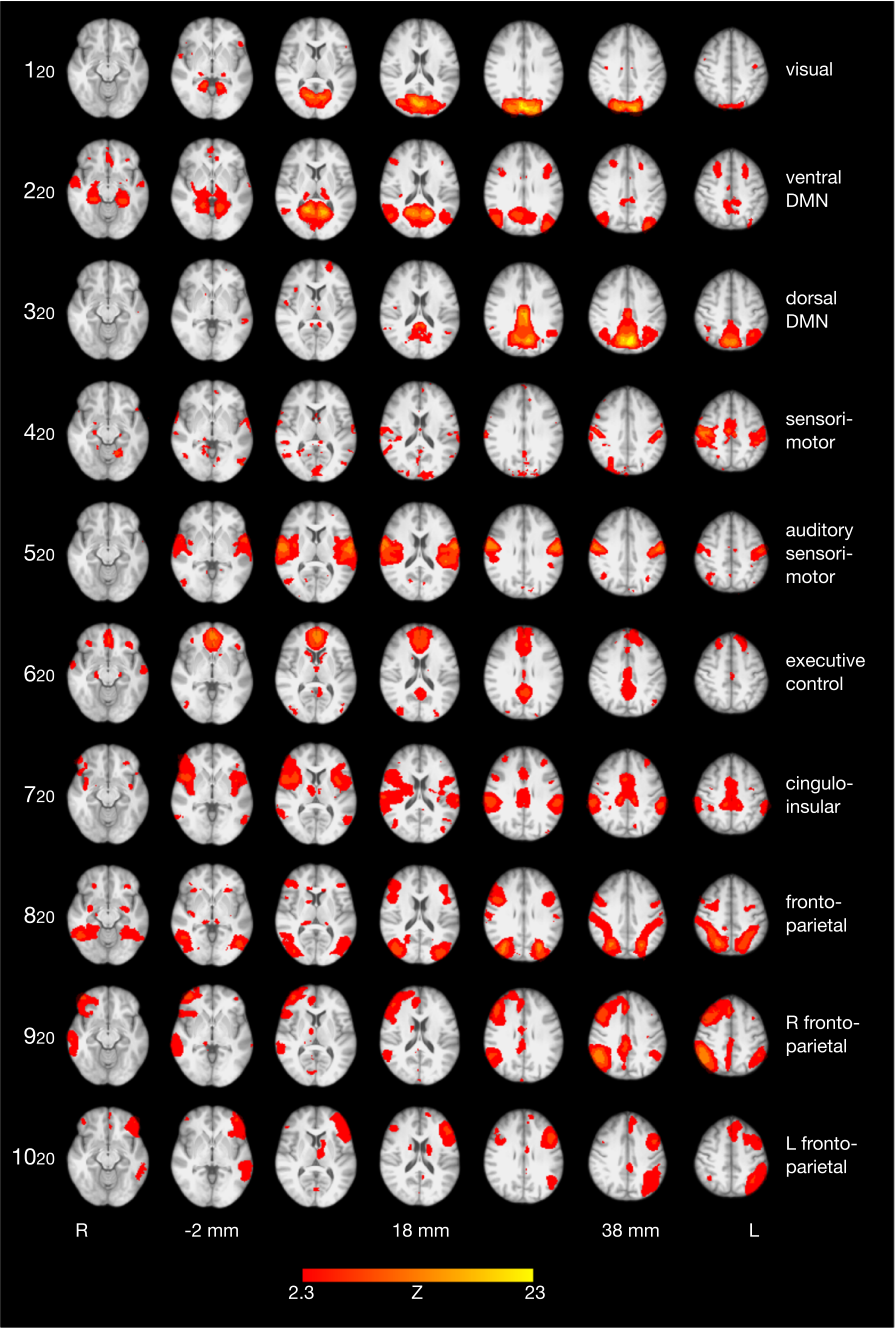
Ten resting state networks (RSNs) identified in a sample of 38 adults with ADHD, corresponding to the 10 RSNs found by Smith et al. [10]. Brain images are displayed in radiological convention (the right hemisphere appears on the left side of the image)

After low-dimensional decomposition with MELODIC, the following RSNs, described by Smith et al. [10], were also found in our sample: the visual, default mode, sensorimotor, auditory, executive control, and bilateral fronto-parietal networks. The DMN, one RSN in the study by Smith et al. [10], is decomposed into two networks in our analysis, a ventral and a dorsal DMN. The cerebellar RSN, found by Smith et al., did not appear in our study because of the incomplete coverage of the cerebellum during rs-fMRI scanning. Figure 3 illustrates the 10 RSNs identified in our sample of adults with ADHD (components 1–8, 10, 13 of the original 20-component group ICA).

Additional file 1 Appendix 1 summarizes the 10 components that were excluded from further analyses (components 9, 11, 12, 14–20 of the original 20-component group ICA). Of those, component 9 is truncated because of incomplete coverage of the cerebellum. Component 11 shows strong activation of the anterior cingulate, similar to the executive control network included in further analysis (component 6 in Fig. 2). Component 12 shows strong activation in the bilateral inferior frontal and temporal lobes, similar to the auditory and fronto-parietal networks included in further analysis (components 4, 9 and 10 in Fig. 2). The remaining components display primarily artifactual signal changes (i.e., non-neuronal noise).

### Differences in functional connectivity between women and men with ADHD

An independent t-test with age as covariate of no interest did not reveal significant differences in functional connectivity between women and men in our sample.

### Associations between functional connectivity and age

**Fig. 4:**
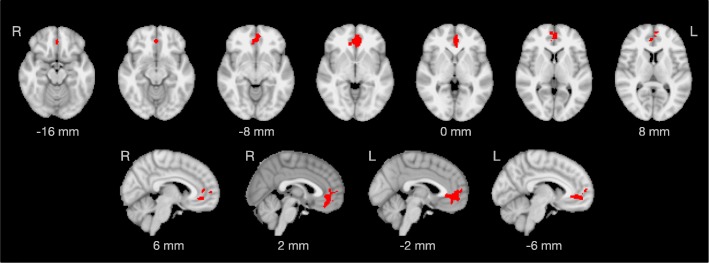
Regions of decreased functional connectivity in older individuals with ADHD within the executive control network (IC 6 in Fig. 2). The significant cluster covers parts of the bilateral anterior cingulate cortex and the bilateral paracingulate cortex (cluster size: 493 voxels). The coordinates of the voxel with highest significance are: x = − 2 mm, y = 42 mm, z = − 2 mm (*p* = 0.004). Brain images are displayed in radiological convention (the right hemisphere appears on the left side of the image).

In ADHD participants with advanced age, a significant decrease of functional connectivity was found in the executive control network (IC 6), covering parts of the bilateral anterior cingulate cortex and the bilateral paracingulate cortex (Fig. 4).

### Associations between functional connectivity and ADHD symptom severity

**Fig. 5:**
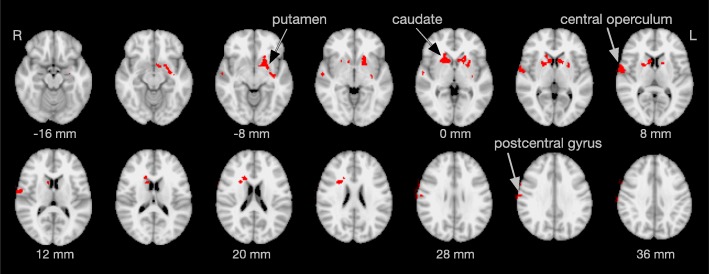
Regions of increased functional connectivity in adults with ADHD with higher scores on the CAARS hyperactivity/restlessness subscale within the auditory / sensorimotor resting state network. Location, *p*-values and cluster sizes are summarized in Table 2. Brain images are displayed in radiological convention (the right hemisphere appears on the left side of the image)

In ADHD participants with higher scores on the CAARS hyperactivity/restlessness subscale, increased functional connectivity was found within the auditory/sensorimotor RSN (IC 5). These areas cover parts of the left putamen, right caudate nucleus, right central operculum and a portion of the right postcentral gyrus (Fig. 5, Table 2). Scores on the inattention/memory problems and impulsivity/emotional lability subscales were not associated with changes in functional connectivity.

**Table 2:** Regions of increased functional connectivity in adults with ADHD with higher scores on the hyperactivity/restlessness subscale

Region	No. of voxels	x (mm)	y (mm)	z (mm)	*p*-value
L putamen	461	−19.3	6.4	−4.3	0.009
R caudate	372	17.2	13.6	8.4	0.011
L postcentral gyrus	235	67.4	−4.3	31.2	0.019
R central operculum	224	61.7	−3.8	6.3	0.014

The x, y, and z coordinates represent the center of gravity of the entire cluster

## Discussion

This study on 38 methylphenidate-naïve adults with ADHD has three main findings. First, on the group level, decomposition of resting state brain activity of adults with ADHD resulted in similar RSNs as previously described for healthy adults [10]. Second, advanced age was associated with decreased functional connectivity in parts of the bilateral cingulate and paracingulate cortex within the executive control network. Third, higher scores on the CAARS hyperactivity/restlessness subscale were associated with increased functional connectivity in cortical and subcortical areas within the auditory/sensorimotor network.

### Resting state networks in adult ADHD

The RSNs found in our ADHD sample demonstrated a close correspondence to previously published RSNs in healthy adults [10]. The RSNs by Smith et al. [10] were derived from a group of 36 healthy individuals (15 women), similar in size to our sample. Moreover, rs-fMRI measurements by Smith et al. [10] and for our study were performed with identical scanner hardware (a 3 T Siemens Magnetom Trio with a 12-channel head coil).

### Decreased functional connectivity with advanced age in adult ADHD

To the best of our knowledge, the association between functional connectivity and age has not been investigated in adults with ADHD before. Here, we found a decrease of functional connectivity in the bilateral cingulate and paracingulate cortices within the executive control network in older ADHD participants (Fig. 4). In a previous study, we analyzed the structural MRIs acquired for this trial (*n* = 64) to determine cortical thickness and subcortical gray matter volumes using surface-based morphometry and subcortical segmentation as implemented in FreeSurfer [23]. Interestingly, we found wide-spread cortical thinning and subcortical volume reduction associated with aging in adults with ADHD, but no significant decrease of cortical thickness in the bilateral cingulate and paracingulate cortices. These structural findings suggest that the age-related decrease in functional connectivity is a genuine phenomenon of brain function and not an epiphenomenon of gray matter loss.

Changes in functional connectivity as a function of age have been described previously in healthy aging [47, 48]. Thus, the observed decrease of functional connectivity in the bilateral cingulate and paracingulate cortex may be specific to ADHD or may be caused by unspecific aging processes. Of note, we did not find a decrease of functional connectivity in the default mode network in older individuals with ADHD. In healthy aging, a decrease of default mode connectivity is a widely replicated finding [47, 48].

### Increased functional connectivity with higher scores on the hyperactivity/restlessness subscale in adult ADHD

We also found increased functional connectivity in parts of the bilateral striatum (in particular, left putamen and right caudate nucleus), right central operculum and right postcentral gyrus in participants with higher scores on the CAARS hyperactivity/restlessness subscale (Fig. 5). The basal ganglia are a set of subcortical nuclei that subserve motor control, various cognitive functions and emotional processing, with the striatum being the primary input nucleus [49]. More recently, evidence has accumulated that the basal ganglia are also involved in behavioral and neural inhibition in motor and non-motor functions [50].

Dysfunction of the basal ganglia and fronto-striatal circuits has long been suggested to be one of the core pathomechanisms of ADHD. This notion has been supported by the dopaminergic effects of methylphenidate, the major pharmacological treatment for ADHD. The reduction of subcortical gray matter in children with ADHD [4–6] appears to normalize in adults [6] and is probably not involved in basal ganglia dysfunction in adults with ADHD. In a recent analysis of the structural MRIs of more than 500 adults with ADHD (> 21 years) and more than 400 healthy controls, no significant difference in volume was found for any of the subcortical nuclei under investigation, including the caudate nucleus, putamen and pallidum [6].

Functional MRI of inhibitory control in adult ADHD led to inconsistent results [51]. Sebastian et al. [52] performed fMRI in stimulant-naïve adults with ADHD during three different experimental tasks probing interference inhibition, action withholding and action cancelation. This study disclosed hypoactivation of the basal ganglia during action withholding and action cancelation [52]. By contrast, a quantitative meta-analysis of fMRI studies on inhibitory control in 100 adults with ADHD in total (including individuals receiving long-term stimulant medication) concluded that adults with ADHD have hypoactivation of the right inferior frontal cortex and right thalamus relative to controls, but no hypoactivation of the basal ganglia [53].

A recent study by Mostert et al. [28] compared rs-fMRI in 99 adults with ADHD with 113 healthy individuals and found stronger functional connectivity in the anterior cingulate gyrus of the executive control RSN, but no differences in connectivity in the basal ganglia or the default mode network.

The aforementioned studies performed categorical comparisons between individuals with ADHD and healthy controls. ADHD, however, is characterized by a remarkable phenotypic and genetic heterogeneity [54, 55] and comparisons on the group level may fail to uncover neural dysfunction in heterogeneous ADHD samples. For this reason, we performed dimensional analyses with the individual levels of symptom severity as continuous regressors [56] which enabled us to detect associations between a clinical parameter (hyperactivity/restlessness) and resting state brain activity.

In a large sample of adolescents with ADHD, Oldehinkel et al. [57] performed both categorical and dimensional analyses of functional connectivity in striatal networks. Comparing 169 adolescents with ADHD and 122 healthy individuals did not reveal functional differences in striatal networks. A dimensional analysis, by contrast, demonstrated an association between increased hyperactivity/impulsivity scores and increased inattention scores with increased functional connectivity in the networks of posterior putamen and ventral caudate [57]. Similarly, in children with the hyperactive-impulsive subtype of ADHD, increased connectivity in the cortico-striatal network was found, whereas children with the inattentive subtype showed increased connectivity in the ventral attention network [58]. The results of the present study corroborate the findings of Oldehinkel et al. [57] and Sanefuji et al. [58]. In summary, a dimensional approach may be better suited to identify changes in basal ganglia connectivity [57, 58] than a categorical approach [28].

Contrary to our initial hypothesis, no significant associations between functional connectivity within the ventral and dorsal DMNs and the symptom severity scores were identified in the present study. This result is also in contrast to previous studies in childhood, adolescent and adult ADHD, describing weaker connectivity within the DMN in individuals with ADHD vs. controls [8]. In adult ADHD, decreased functional connectivity was found between the anterior cingulate and the precuneus/posterior cingulate cortex regions in a seed-based analysis [13] and between the precuneus and other areas of the DMN using a network homogeneity analysis [59]. By contrast, the large study on resting state functional connectivity by Mostert et al. consisting of 99 adults with ADHD, using group ICA and dual regression very similar to the present study, did not find differences in DMN connectivity between adults with ADHD and controls. Taken together, the involvement of the DMN in adult ADHD is not well established. Future studies are needed with larger sample sizes, comparing and integrating the results of different analysis strategies.

### Strengths and limitations

Our study comprises a clinically-well characterized sample of 38 methylphenidate-naïve adults with ADHD. Many studies in the field include participants with long-term stimulant medication as well (and discontinue medication ~ 24–48 h before fMRI, e.g. [28, 57]. Investigating a stimulant-naïve sample is beneficial because of potential effects of long-term stimulant medication on brain structure and function [60, 61]. Another strength of our study is a stringent two-step head motion correction with a standard motion correction with volume-realignment and an additional ICA-based de-noising of the preprocessed data sets.

Limitations of our study are the absence of a healthy control group, which prevented us from performing categorical comparisons between adults with ADHD and healthy individuals. Moreover, the fMRI scans used for this study did not cover the entire cerebellum, a structure, which has been implicated in the pathogenesis of ADHD [62].

Future studies of resting state brain activity in ADHD should make use of advanced imaging techniques that allow a considerable reduction of the TR (simultaneous multislice imaging) [63].

## Conclusions

This study corroborates and extends our knowledge on the involvement of the striatum in the pathophysiology of ADHD, in particular, in the pathogenesis of hyperactivity. Moreover, we found, for the first time, a decrease of functional connectivity in the bilateral cingulate and paracingulate cortices within the executive control network in older individuals with ADHD. Significant associations between functional connectivity in the default mode network and symptom severity, sex or age were not found. Our results emphasize the usefulness of dimensional analyses with individual symptom severity and age as regressors in the study of ADHD, a highly heterogeneous disorder.

## Additional file

Additional file 1: **Appendix 1.** Overview of the 10 independent components that were not used for further analysis. (PDF 309 kb)

## Abbreviations

ADHD: Attention-deficit/hyperactivity disorder
BET: Brain extraction tool
BOLD: Blood oxygenation level dependent
CAARS: Conners adult ADHD rating scales
DMN: Default mode network
DSM-IV: Diagnostic and statistical manual of mental disorders, 4th edition
FLIRT: FMRIB’s linear image registration tool
fMRI: Functional magnetic resonance imaging
FNIRT: FMRIB’s non-linear image registration tool
FoV: Field of view
FSL: FMRIB’s Software Library
FWE: Family-wise error
IC: Independent component
ICA: Independent component analysis
ICA-AROMA: Independent component analysis-based automatic removal of motion artifacts
MCFLIRT: Motion correction FMRIB’s linear image registration tool
MELODIC: Multivariate exploratory linear decomposition into independent components
MP-RAGE: Magnetization-prepared rapid acquisition gradient-echo
rs-fMRI: Resting state functional magnetic resonance imaging
RSN: Resting state network
TE: Echo time
TFCE: Threshold-free cluster enhancement
TI: Inversion time
TR: Repetition time

## Footnotes

1 http://fcon_1000.projects.nitrc.org/indi/adhd200/

2
https://www.isrctn.com

3
10.1186/ISRCTN12722296

4
http://www.fmrib.ox.ac.uk/fsl

5
https://github.com/rhr-pruim/ICA-AROMA

6
https://fsl.fmrib.ox.ac.uk/fsl/fslwiki/FNIRT

7
https://www.fmrib.ox.ac.uk/datasets/brainmap+rsns
