# Supplementary figures and images for: Hyperactivity/restlessness is associated with increased functional connectivity in adults with ADHD: a dimensional analysis of resting state fMRI
Source: BMC Psychiatry. 2019 Jan 25;19:43. doi: 10.1186/s12888-019-2031-9 (PMC6347794; doi:10.1186/s12888-019-2031-9)

R

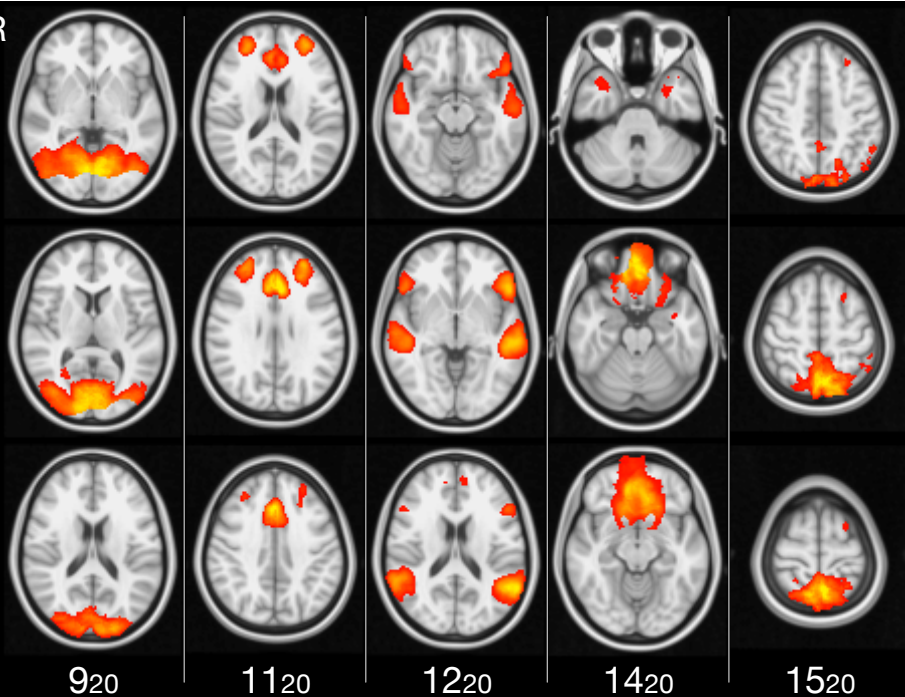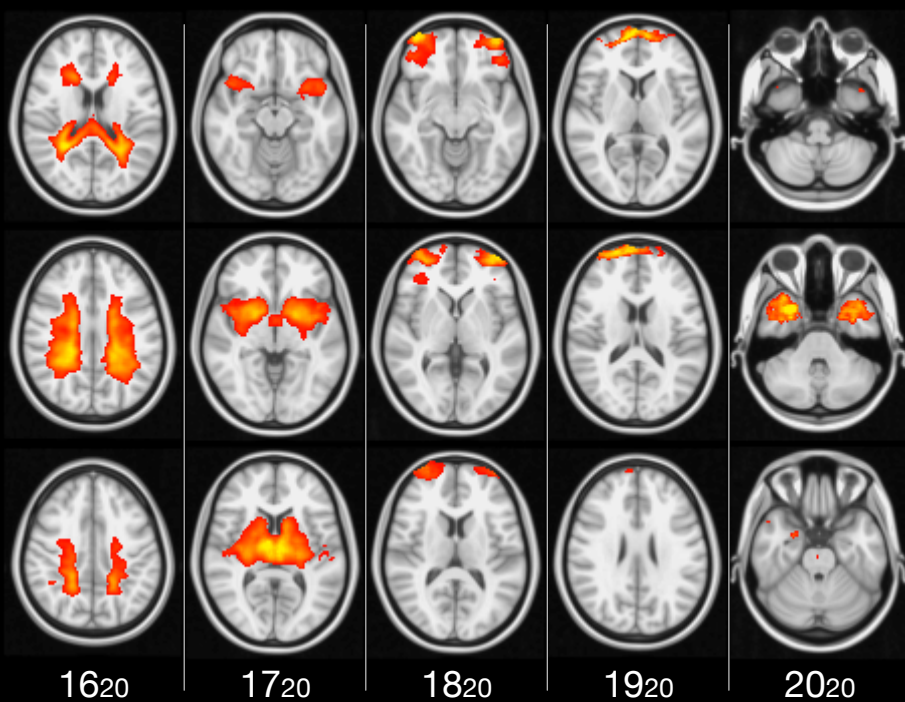

Supplement: Supplementary file 1 — Appendix 1. Overview of the 10 independent components that were not used for further analysis. (PDF 309 kb) [file 12888_2019_2031_MOESM1_ESM.pdf]
